# At the Deathbed of Consumptive Art

**DOI:** 10.3201/eid0811.020549

**Published:** 2002-11

**Authors:** David M. Morens

**Affiliations:** University of Hawaii School of Medicine, Honolulu, Hawaii, USA

**Keywords:** history, infectious diseases, public health, tuberculosis

**Figure:**
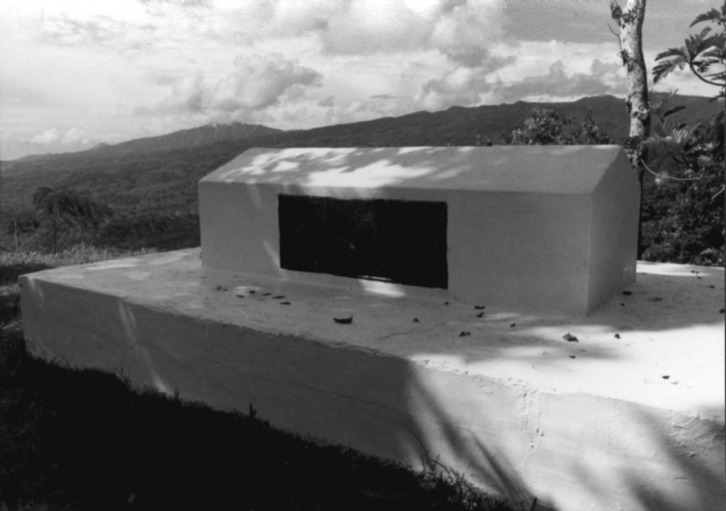
Robert Louis Stevenson's sarcophagus, on top of Mount Vaea, Upolu, Western Samoa. Photo courtesy of David Morens.

Under the wide and starry sky,
Dig the grave and let me lie.

R. L. Stevenson, “Requiem”

For more than a century, readers have pondered the strange beginning to one of the most haunting poems in the English language, “Requiem.” Who has not wondered how a poet can seem to welcome his own death? Scotsman Robert Louis Stevenson died of a disease so poorly understood in his day that over a few decades its preferred name changed three times, from "phthisis" to "consumption" to "tuberculosis.” A century later, we have reliable scientific facts on this "old" poet-killing disease—we know for one that *Mycobacterium tuberculosis* now infects 1.9 billion people, nearly a third of the world’s population (nearly 2 million deaths each year). But human suffering is still difficult to quantify. 

Two million deaths each year. How can we grasp such statistics of misery? Reduced to numbers stacked up in columns and cut up in pie charts, tuberculosis patients don’t seem like us. They live in faraway places, come from obscure cultures, speak incomprehensible languages, have disreputable comorbid disease, or exhibit antisocial behavior. They are “the other.” Maybe they do not even exist.

To grasp the human suffering perpetrated by tuberculosis, we may need to recall the past when, incurable and incomprehensible, the disease had to be deciphered by metaphors—metaphors that changed as societal views of the disease changed over time. We may need to recall the lives of dying artists and the work they created and let art paint their faces, sculpt their shapes and contours, and compose leitmotivs. Perhaps such *past *images will help fix the gazes of *today’s *victims, whose faces we do not seem to be able to see. 

The arts (the novel, the play, the poem, the musical composition, the operatic production) have always helped us understand, given us perspective, invoked compassion, and argued a purpose and meaning for life. We listened to the molto adagio of Barber’s string quartet (opus 11), and came together at President Roosevelt’s death. We read the poems of Walt Whitman and taught our children about the soldier's sacrifice. We studied the works of Petrarcha and Guillaume de Machaut and revisited the terror of Black Death, 700 years ago. Now, we must turn to art again to grasp human suffering because scientific knowledge of the disease seems to have displaced our interest in the patient. 

## Tuberculosis and the Arts

### Poetry

In his modest room, a 25-year-old "lapsed" medical student in 1820s Great Britain wakes from a sudden fevered sweat and finds a single drop of blood on the sheet. He has known many patients who spit such bright blood. “It’s arterial blood…that blood is my death warrant, I must die,” he confides to a friend. One of England’s greatest poets, the medical student John Keats, never wrote specifically about phthisis. But his life and his works became a metaphor for generations of patients, a metaphor that helped transform the physical disease phthisis” into its spiritual offspring, consumption. 

Keats’ life was defined by tuberculosis. At 14, he nursed his mother as she died of phthisis. A few years later he watched his brother die of phthisis, and by age 23, he had symptoms of this “hereditary ailment” himself. Yet, as the best remedies (bleeding, diet, red wine, opium) failed, as his work was savaged by critics and his love was languishing (he could not marry because of the disease), Keats feverishly wrote his greatest poems: Ode to a Nightingale, Ode on a Grecian Urn, Ode on Melancholy. 

He died only months after he first spit blood. Autopsy found his lungs completely destroyed. He was 26. To be dead himself within the year, Percy Bysshe Shelley, another young poet with phthisis, compared Keats to “a pale flower by some sad maiden cherished,/And fed with true-love tears, instead of dew/The bloom, whose petals nipped before they blew/Died on the promise of the fruit, is waste;/The broken lily lies—the storm is overpast.”

Shelley’s tribute expressed what would become the central metaphor of consumption in the 19th century, the idea that the phthisic body is consumed from within by its passions, “the bloom…Died on the promise of the fruit.” Shelley also likened Keats to an eagle that “outsoared the shadow of our night,” and “could scale Heaven, and could nourish in the sun’s domain.” These romantic notions contrasted sharply with the actual demise of tuberculosis patients, poets or not. As death neared, Keats himself contradicted his friend. In “On Seeing the Elgin Marbles” (published posthumously), Keats wrote, “My spirit is too weak—mortality/Weighs heavily upon me like unwilling sleep,/And each imagin’d pinnacle and steep/Of godlike hardship, tells me I must die/Like a sick Eagle looking at the sky.”

In succeeding decades, Keats’ illness came to exemplify spes phthisica, a condition believed peculiar to consumptives in which physical wasting led to euphoric flowering of the passionate and creative aspects of the soul. The prosaic human, it was said, became poetic as the body expired from consumption, genius bursting forth from the fevered combustion of ordinary talent, the body burning so that the creative soul could be released. Keats’ great poetic output during his last year was considered a direct consequence of consumption. 

Spes phthisica, which sought to make sense of the senseless and give purpose to purposeless suffering and death, came to be viewed as a prerequisite for creative genius. French author Alexandre Dumas fils wrote, “It was the fashion to suffer from the lungs; everybody was consumptive, poets especially; it was good form to spit blood after any emotion that was at all sensational, and to die before reaching the age of thirty.” Dumas’ colleague, the poet Théophile Gautier, wrote, “…I could not have accepted as a lyrical poet anyone weighing more than ninety-nine pounds.” A subsequent alleged decline in the arts was even blamed on decline in tuberculosis incidence. 

### Opera and the Novel

In the 19th century, it seemed as if everyone was slowly dying of consumption. Without explanation, the good and the bad, the young and the old, all shared the consumptive fate. Consumption came to be viewed not in medical terms (medicine had little to offer anyway), but in popular terms, first as romantic redemption, then as reflection of societal ills. The consumptive prostitute, for example, could be a moral deviant redeemed by suffering and death. Redemption occurred not in the confessional, nor in the "last rites," but in consumption’s physical sacrifice. Alternatively, the prostitute could be merely a woman victimized by a corrupt society.

Opera perhaps provides the most powerful examples of romantic redemption through tuberculosis. The pallor and wasting, the burning sunken eyes, the perspiration-anointed skin—all hallmarks of the disease—came to represent haunted feminine beauty, romantic passion, and fevered sexuality, notions reinforced by the excess of consumption deaths in young women. Of several operas that deal explicitly with consumption, three (written within a 6-year interval but produced successively over the last half of the 19th century) show the evolution in thinking about the disease.

Verdi’s La traviata [The Straying One] is based on La dame aux camélias [The Lady of the Camellias], Dumas’ tale chronicling the life of Parisian beauty Rose Alphonsine Plessis (also known as Marie Duplessis, 1824–1847). Violetta, the heroine, is a courtesan whose loveliness is stereotypically enhanced and made unforgettable by progressive consumption. So strong was the expectation that women dying of consumption be beautiful ghosts that, at La traviata's 1853 premiere, the audience broke out laughing when Violetta, played by the ample soprano Fanny Salvini-Donatelli, was told she had only hours to live. 

The plot of La traviata develops around the consequences of Violetta’s scandalous past, which prevents her marriage to an honorable man whose family objects. In the libretto (by Francesco Maria Piave), Violetta links Alfredo’s acceptance to a cure for her consumption. Referring to their separation, she asks, “Do you know the fatal ill, That attacks my life—to kill? That already draws the end? And you’d part me from my friend!” 

Eventually realizing that redemption is possible only through death, Violetta withdraws to her former world to suffer and die for love: “If he knew the sacrifice Whereby love I atone—Knew that to my last breath I loved just him alone…." In taking her life, consumption also serves as a vehicle for atonement. Violetta dies redeemed in the eyes of Alfredo and his father, her labored breaths fading as she pleads: “Look at me! I breathe for you still.” 

Produced only 28 years later (1881; the year before Robert Koch’s "discovery" of *Mycobacterium tuberculosis*), Les contes d’Hoffmann [Tales of Hoffmann] exhibits an important shift in thinking about consumption. In Hoffmann, the beautiful consumptive Antonia is treated by the charlatan physician Dr. Miracle. At the time of Keats’ death, in 1821, little could be done to treat phthisis; the physician's role was in prognosis. By 1881, the “medicalization” of consumption was in full swing, with diets, nostrums, regimens, and activity lists (all of little or no benefit). 

By portraying Antonia as a victim of quackery, Hoffmann satirizes medical impotence. Further, the opera links Antonia’s consumption to her mother’s. Heredity as a possible cause of consumption (a popular concept before Koch’s discovery) appealed to the opera’s audience because it absolved the patient from guilt or shame. Heredity as cause may have also appealed to the opera’s composer Jacques Offenbach—both he and his son were consumptive. The libretto (by Jules Barbier and Michel Carré) seems also to presage a new medical concept, a mycobacterial neurotoxin postulated as the cause of spes phthisica after Koch’s discovery. 

In Hoffmann, Dr. Miracle asks, “Well, this trouble she inherited From her mother? Still progressing. We’ll cure her.” As he forces her to sing, in what amounts to a musical exorcism, he notes, “The pulse is unequal and fast, bad symptom [sic]. Sing.” The ghost of Antonia’s mother, conjured by Dr. Miracle, calls to her, and she sings frantically, an artistic representation of her consumptive decline. Nearing death, she asks, “What ardor draws and devours me? I give way to a transport that maddens. What flame is it dazzles my eyes. A single moment to live, And my soul flies to Heaven.” Antonia is merely a victim, not burdened by sins that need to be atoned for. Unlike Violetta, she dies maddened by disease, without comprehending why and without making any moral choice.

By 1896, the cause of consumption had been discovered. Tuberculosis or TB (as the disease was now known to everyone) had also been definitively linked to poverty and industrial blight, child labor, and sweatshops. A contagious disease and shameful indicator of class, it was no longer easily romanticized in conventional or artistic terms. Public health efforts to isolate the infected and control their behavior were everywhere. 

Giacomo Puccini’s La bohème (1896) presents tuberculosis as social commentary and features characters new to opera, street artists living with poverty and disease. The opera was fashioned from Bohemians of the Latin Quarter by Louis-Henri Murger, a young writer who also died of tuberculosis. The heroine, Mimì, is based on Murger’s friend Lucille Louvet. A poor seamstress portrayed as a fevered beauty whose allure is heightened by physical decline, Mimì loves a poor poet named Rodolfo. He first loves her in return but then abandons her, fearing he cannot provide for her and (possibly) fearing contagion. 

In the libretto (by Giuseppe Giacosa and Luigi Illica), Mimì tells Rodolfo, “My story is brief. I embroider silk and satin at home or outside. I love all things that have gentle magic, that talk of love, of spring…the things called poetry. Do you understand me? They call me Mimì. I don’t know why. I live all by myself and I eat alone. I look at the roofs and the sky. But when spring comes the sun’s first rays are mine. April’s first kiss is mine, is mine! A rose blooms in my vase, I breathe its perfume, petal by petal.” 

Later, in the winter cold, Mimì is reconciled with Rodolfo and dies beside him. In one of opera’s most enduring scenes, there is no attempt at metaphorical understanding. Mimì dies literally. No one is saved, no one is redeemed, and no larger point is made. Opera and tuberculosis have entered a new era, recognizable today, in which tragedy is seen as experiencing loss but is not understood in an artistic or philosophical sense. In the moments before her death, Rodolfo tells Mimì that she is “beautiful as the dawn.” “You’ve mistaken the image:” she corrects him, “You should have said, beautiful as the sunset.” Mimì dies surrounded by a philosopher, a poet, a painter, a musician, and a singer—the arts had become powerless against tuberculosis.

Even though new, La bohème’s approach of pointing the finger at society as the cause of human suffering was not unprecedented. An earlier example from literature is Victor Hugo’s Les misérables [The Outcasts], published in 1862. In the novel, the hounded protagonist (Jean Valjean) finds redemption in the child of a dying woman (Fantine) who had been forced by poverty into prostitution. Like Mimì’s in La bohème, Fantine’s death from consumption is portrayed as a consequence of social ills. Society, Hugo says, victimizes good people by putting them in hopeless circumstances. 

Of Fantine’s death Hugo writes with a simple beauty that is eloquent even in translation. Like the “afterthought” the world had regarded the poor woman’s entire life, the paragraph describing her death is inserted almost randomly, in retrospect: “We all have one mother—the earth. Fantine was restored to this mother. [She] was buried in the common grave of the cemetery, which is for everybody and for all, and in which the poor are lost. Happily, God knows where to find the soul. Fantine was laid away in the darkness with bodies which had no name; she suffered the promiscuity of dust. She was thrown into the public pit. Her tomb was like her bed.” 

In the same romantic context is Hugo’s earlier novel Notre-Dame de Paris (1831), which featured the grotesque and deformed “hunchback” Quasimodo. In those days, the principal cause of severe spinal deformity was Pott’s disease, tuberculosis of the spine. What Hugo knew of this condition can only be speculated, but as a leading poet, he would unquestionably have been familiar with the life of one of England’s greatest poets, Alexander Pope (1688–1744), a hunchback severely deformed in childhood by Pott’s disease. Bent to a height of 4½ feet, at age 47, Pope famously described “this long disease, my life” in “An Epistle from Mr. Pope, to Dr. Arbuthnot.” Quasimodo’s physical “otherness” is Hugo’s device for insisting we recognize his humanity, without judgment, in all its dimensions, good and ill. 

Notre-Dame de Paris carries a simple message, one expunged from many films and operas made of the book. Hugo writes that long after the events depicted in the novel, workmen discovered two entwined skeletons, one of a young woman, the other of a man with a twisted spine. After La Esmeralda’s execution, the reader surmises, Quasimodo had gone to end his life beside the woman who had found him so repugnant. In death, his disfigurement and her beauty alike were dissolved in dust. All of Quasimodo’s actions, misunderstood by everyone else, are explained by love, as pure in the deformed, slow-witted bell-ringer as in the beautiful saint.

Operatic and literary examples show the romantic transformation of tuberculosis. Only rarely in the period before Koch's discovery was the disease portrayed realistically in artistic works. The most powerful example of realistic portrayal may be Tolstoy's Anna Karénina (1877). Apparently based on Tolstoy's own brother Nikolai, the novel’s character Nikolai Levin suffers an agonizing and vividly depicted death. "A desire for his death was now felt by everyone who saw him; by the hotel servants and the proprietor…by Maria Nikolayevna, Levin, and Kitty. [I]n rare moments, when opium made him find momentary oblivion…[he] expressed what he felt more intensely in his heart than all the others: ‘Oh, if only it was all over!’ or ‘When will this end?’ There was no position in which he could lie without pain, there was not a moment in which he could forget…And that was why all his desires were merged into one—a desire to get rid of all the sufferings and their source, the body." Unlike the prostitute whose death provided Jean Valjean's redemption, Maria was rescued from prostitution by Nikolai and nursed him when he was abandoned. As Nikolai entered the long descent into suffering and death, Maria's love was his only human connection.

## Science and Public Health

### The Romantic Era of Consumption (1821–1881)

At the beginning of this period, the stethoscope was invented and used to diagnose phthisis, and statistics were compiled by population-oriented proto-epidemiologists (e.g., Louis-François Benoiston de Châteauneuf) and by clinical proto-epidemiologists (e.g., Pierre-Charles-Alexandre Louis). In the 1820s, contagion was beginning to coalesce into a modern concept, although it was not imagined in chronic conditions like phthisis. Science was beginning to understand the disease clinically and epidemiologically (not yet etiologically). It was left to the arts to make sense of misery and death in ways that turned otherwise senseless suffering into human dignity and hope, allowing consumption to reveal the innate worth even of prostitutes, the impoverished, and the socially outcast. 

### Discovery of *Mycobacterium tuberculosis *(1882–1952)

At the end of the 19th century, romantic notions about tuberculosis were replaced by scientific ideas and products: vaccines and therapies, rest cures, tonics, lobectomies, pneumonectomies, thoracoplasties, “artificial” pneumothoracies, phrenic nerve crushings, plombage, pneumatic cabinet treatments, and antiseptic injections into the pleural spaces. Surgeons packed the pleural cavity with fat, paraffin, and even Ping-Pong balls. In contrast to the arts, science and medicine were unapologetically prosaic.

Over a 70-year span, tuberculosis literally transformed Western society. Tuberculosis patients were excluded from many occupations. Married patients had to sleep in separate beds from uninfected spouses and were counseled to avoid sex and especially to not have children. Public health nurses visited door to door, sanatoria were built by the hundreds, and hospitals added tuberculosis wings. Cold water hydrotherapy, alcohol massages, and brisk rubdowns with coarse towels were prescribed. Millions of spittoons and cuspidors were placed in homes and public places. Patients’ bed linens were changed daily and were boiled and laundered separately. Handkerchiefs (for those who could afford them—cut-up pieces of muslin for the rest) were stuffed in pockets before leaving home, then disinfected or burned at night. Japanese “paper handkerchiefs” became popular, leading eventually to the modern “facial tissue.” Tuberculous women had to forego corsets and brassieres in favor of loose-fitting clothes. Life insurance policies added clauses canceling benefits for the tubercular, and hotels and landlords refused to serve them. Compulsory registration, immigration bans, and even interstate travel restrictions were debated. Suicides in towns with sanatoria increased. 

Prevention efforts were put in place. National antituberculosis programs were led by U.S. presidents. Ubiquitous “ad campaigns” featured catchy tunes some called “jingles.” Architects designed alcoves to hide concealed spittoons in middle-class homes. Babies were no longer allowed to play on the floor, and mothers were told not to kiss children on the mouth. Some churches abandoned the “common” communion cup. "TB" and “x-ray” became household words. Streets were watered down before sweeping to prevent aerosols. Long “trailing” dresses went out of fashion because they dragged on the ground and picked up potentially infectious dust. Store candies and bakery loaves had to be wrapped; public libraries and their books were regularly disinfected. 

### Modern Times (1952–2002)

In the late 1940s and early 1950s, a truck cycled through neighborhoods to administer chest x-rays, and schools provided “tine tests.” Patients sat in big sunrooms off hospital wards, semi-erect in lounge chairs, wrapped like mummies on an ocean cruise. Family members disappeared for long periods—off to sanatoria for “rest cures.” People bought Christmas Seals, and spoke in a slight hush when they mentioned “TB.” As a medical student in the 1960s, I remember the second patient I ever examined. Born in 1879, she had undergone a right phrenic nerve crush, sometime around 1910, surviving tuberculosis to the age of 90. 

The 1950s represented the cusp of a new era in which drug treatment would end tuberculosis visibility in industrialized countries. People still read Thomas Mann’s The Magic Mountain (1924), which was set in the real-life sanatorium in Davos, Switzerland, but less to learn about tuberculosis and more to understand the “Sanatorium Movement.” The modern tuberculosis era began around 1952 with antituberculosis chemotherapy, but more die of the disease today than in the 19th century. Medicine and public health have answers but lack the will to apply them. Tragedy now derives not from unknown evils out of our control but from known evils we do not control. 

Art is redemptive. Even in modern times, when artistic expression seems to have moved to other causes of human suffering, occasionally dramatic irony sneaks backstage to comment on the empty theater of tuberculosis progress. In 1967, a woman born in India, acclaimed a great actress in such roles as Anna Karénina and Scarlett O’Hara (Gone with the Wind), died of tuberculosis. Starring at the time in a long-running play, Vivian Hartley (stage name Vivian Leigh) began to experience cough, then fever. Within a few months she was dead. The accidental irony was pure “theater.” The play was Chekhov’s Ivanov. Vivian Leigh, a wealthy woman dying of tuberculosis, was starring in a play about a wealthy woman dying of tuberculosis, written by a wealthy physician dying of tuberculosis. One may imagine that when art fails to imitate life, life may have no recourse but to imitate art.

## Odysseys

 In the early 1980s, I realized a boyhood dream, to climb an isolated peak called Mount Vaea, on the remote South Pacific island of Upolu, in what is now Western Samoa. Around that time, one of my patients, born on a Pacific island and admitted to my American hospital for tuberculosis, had walked up to the nursing station for some trivial purpose and in the middle of a conversation with the nurse, had suddenly opened his mouth to an explosion of arterial blood that shot across the room, overflowed like a waterfall to the linoleum floor, and caught him in its plumes as he sank downward and died—suffocated or drowned. 

A century earlier, in 1880, coughing blood and dying more slowly, Robert Louis Stevenson fled his consumptive sentence. He escaped to Davos, to rugged sea-swept Speyside, and to sunny Marseille. All the while, he worked on the great books we still read all over the world, Treasure Island, Kidnapped—novels of escape. 

Surviving a succession of hemorrhages, Stevenson climbed hills and sailed oceans, following the medical advice of his time. In New York, he stayed at Saranac Lake, America’s most famous sanatorium. In San Francisco, he was farther from home than he had ever been. Still the hemorrhages and wasting continued. Escaping ever farther, he went to Hawaii, lived in a grass hut, and told stories to the King and Queen. He went on to Sydney, Australia, where he had additional hemorrhages, and at last to Upolu. Tuberculosis nevertheless continued to consume him. He wrote every day, suffering sudden hemorrhages, waiting to be “weaned from the passion of life.” 

The doctors said it was a stroke that felled him in December 1894. The 44-year-old Stevenson was placed on a bed in the middle of the room. Family and friends beside him, his Samoan “family” kneeling in a circle all around, the great Tusitala, “teller of tales,” took a last breath. A council of chiefs was called, appointing 40 strong men to carry out Stevenson’s final wish. They carried his body up to the top of the highest mountain and buried him in the place he had chosen.

Although I have visited the place several times—once dragging a famous epidemiologist barefoot up the mountain—it was the first time, 20 years ago, that I remember most clearly. I reflected then that in 1891, when Stevenson had escaped to Upolu, tuberculosis was still a disease of wealthy industrial nations. To run from it, Stevenson had to leave behind everything else in his life: family, friends, mother country, home, comfort. He fled to the very end of the earth. 

Now, more than a century later, tuberculosis has escaped to the places where its victims once sought refuge, the one-time colonies of Western nations. The disease destroys the poor and underprivileged as it once destroyed the wealthy—95% of cases and 98% of deaths occur in the developing world. Its ravages are worst in the Pacific Islands. I wonder if the victims, who are too often beyond the reach of Western cures, have their own operas, poems, or plays to explain their predicament. I wonder where they escape. Do their escapes, like Stevenson's, lead only to resignation and surrender? 

On top of Mount Vaea, at 1,500 feet, a small promontory shows the blue Pacific in a broad sweep. Facing “Magic Mountain” half a world away, you see a blue curtain above and below the horizon. At night, the sky is indeed wide and starry. The Southern Cross sparkles wildly, too low and too close to be celestial. The sarcophagus, placed at the highest point, is of white plaster, like the walls of the European hospitals and sanatoria Stevenson had fled. The plaque is of shiny brass, so that even in the moonlight, sometimes even in the tropical starlight alone, it is possible to read the words engraved on the side of the tomb. It is Stevenson’s “Requiem,” the poetry of his last resigned step in a long but unsuccessful flight.

Under the wide and starry sky,
Dig the grave and let me lie.
Glad did I live and gladly die,
And I laid me down with a will.
This be the verse you grave for me:
“Here he lies where he longed to be;
Home is the sailor, home from the sea,
And the hunter home from the hill.”

### Acknowledgments
